# *IL-10* Gene Polymorphisms and Susceptibility to Systemic Lupus Erythematosus: A Meta-Analysis

**DOI:** 10.1371/journal.pone.0069547

**Published:** 2013-07-23

**Authors:** Ping Liu, Jianwen Song, Hui Su, Linli Li, Ning Lu, Rongli Yang, Zhenhui Peng

**Affiliations:** 1 Department of Dermatology, Second Affiliated Hospital, School of Medicine, Xi’an Jiaotong University, Xi’an, Shaanxi, China; 2 Department of Dermatology, Xi’an Children’s Hospital, Xi’an, Shaanxi, China; 3 Department of Dermatology, Central Hospital, Shijiazhuang, Hebei, China; 4 Department of Dermatology, First Hospital, Baoding, Hebei, China; Université Libre de Bruxelles, Belgium

## Abstract

**Background:**

A number of observational studies have been conducted to investigate the association of the *IL-10* gene polymorphisms with systemic lupus erythematosus (SLE) susceptibility. However, their results are conflicting.

**Method:**

We searched published case-control studies on the *IL-10* polymorphisms and SLE in PubMed, EMBASE and Chinese Biomedical Literature Database. A meta-analysis was conducted using a fixed-effect or random-effect model based on between-study heterogeneity.

**Results:**

A total of 42 studies with 7948 cases and 11866 controls were included in this meta-analysis. Among Caucasians, the CA27 allele of the *IL10.G* microsatellites (OR 2.38, 95% CI 1.01–5.62), the G allele of the *IL-10* -1082G/A polymorphism (G vs. A: OR 1.21, 95% CI 1.02–1.44; GG vs. AA: OR 1.45, 95% CI 1.16–1.82; GG+GA vs. AA: OR 1.16, 95% CI 1.03–1.29) and its associated haplotype -1082G/−819C/−592C (OR 1.25, 95% CI 1.10–1.42) were associated with increased SLE susceptibility without or with unimportant between-study heterogeneity. Removing studies deviating from Hardy-Weinberg equilibrium (HWE) hardly changed these results. Among Asians, the CA21 allele of the *IL-10.G* microsatellites (OR 1.28, 95% CI 1.02–1.60) and the -1082G/−819C/−592C haplotype (OR 1.24, 95% CI 1.00–1.53) were associated with increased SLE susceptibility, but with substantial between-study heterogeneity or sensitive to HWE status. Removing studies deviating from HWE also produced statistically significant associations of the *IL-10* -1082G/A (GG vs. AA: OR 3.21, 95% CI 1.24–8.28; GG vs. AA+GA: OR 2.85, 95% CI 1.19–6.79) and -592C/A polymorphisms (CC+CA vs. AA: OR 0.69, 95% CI 0.51–0.94) with SLE among Asians.

**Conclusion:**

This meta-analysis showed that the *IL10.G* microsatellites, the *IL-10* -1082G/A and -592C/A polymorphisms and the haplotype -1082G/−819C/−592C are associated with SLE susceptibility. Besides, this is the first time to report an association between the CA27 allele of the *IL-10.G* microsatellites and SLE among Caucasians. Further studies are needed to confirm these findings.

## Introduction

Systemic lupus erythematosus (SLE) is a chronic systemic autoimmune disorder with diverse clinical manifestations. It can be potentially fatal when major organs are affected. The prevalence of SLE ranges from approximately 20 to 150 cases per 100,000 persons worldwide [1], with a female-to-male ratio of 9∶1 [2]. People of Afro-Caribbean and Asian ethnicity are more likely to develop this disorder than white people [3]. Sunlight, drugs and some occupational exposures could trigger the disorder [2]. Infections of Epstein-Barr virus and bacteria have also been identified as possible factors in the development of SLE, but no one specific cause has been identified [2]. A strong familial aggregation has been found in SLE [4]; the concordance rate is higher in monozygotic twins than in dizygotic twins [5]. These facts suggest that genetic factors play a role in the development of SLE.

Interleukin 10 (IL-10), primarily produced by monocytes and lymphocytes, is a multifunctional cytokine in immunoregulation and inflammation. There are several lines of evidence suggesting that the *IL-10* gene is a candidate gene for SLE susceptibility. IL-10 enhances B cell proliferation, differentiation and antibody production, and therefore plays a role in B cell hyperactivity and in increasing production of autoantibodies in SLE [6], [7]. It also inhibits functions of T cells and antigen-presenting cells [8], [9], which in SLE may contribute to impaired cell-mediated immunity. Several studies have found that IL-10 production is high in SLE patients and IL-10 serum level correlates with disease activity [10], [11], [12], [13], [14], [15], [16]. Studies in lupus animal models and humans have shown that anti-IL-10 treatment can decrease disease activity in terms of clinical features and biologic markers [17], [18], [19].

In humans, the *IL-10* gene is located on chromosome 1q and encodes for 5 exons. The *IL-10* promoter is highly polymorphic and in this region two CA-repeat microsatellites (*IL-10.G* and *IL-10.R*) and three single nucleotide polymorphisms (SNPs) at positions −1082, −819, and −592 from the transcription start site, have been identified to correlate with IL-10 production [20]. Haplotypes comprising three SNPs at positions −1082, −819, and −592 have also been found to correlate with IL-10 serum level [20].

Considering the role of IL-10 in SLE and the relationship between the *IL-10* gene polymorphisms and IL-10 production, a number of observational studies have been conducted to investigate the association of the *IL-10* gene polymorphisms with SLE susceptibility. However, their results are conflicting. This can be due to insufficient power, small effect of the *IL-10* gene polymorphisms on SLE susceptibility, and false-positive results. Meta-analysis is a statistical method that can overcome the limitations of individual studies [21]. We therefore performed a meta-analysis to clarify the inconsistency among studies and to establish a comprehensive picture of the association between the *IL-10* gene polymorphisms and SLE susceptibility.

## Methods

### Searching

We searched PubMed, EMBASE and Chinese Biomedical Literature Database for relevant reports without language restriction. The last search update was performed on August 31, 2012. The search strategies were based on the following form: (interleukin-10 or synonyms) AND (“systemic lupus erythematosus” or synonyms). Both thesaurus terms and free text were used. Detailed description of the search strategies can be found in supplementary materials (see Method S1). We also screened references of retrieved articles and relevant reviews for additional studies. Any case-control designed studies were considered eligible if they aimed to investigate the relation between the *IL10* gene polymorphisms and SLE risk, no matter which polymorphisms were studied or whether they provided enough data to calculate odds ratios (ORs). Family-based studies were excluded because of linkage considerations.

Two authors (PL, JS) independently screened all reports by title or abstract for those requiring further retrieval, and then independently reviewed these studies for eligibility. Discrepancies were resolved by group discussion. Following information was extracted using predetermined forms: the first author’s name, year of publication, ethnicity, definition and numbers of cases and controls, genotyping method, frequency of *IL10* genotypes, and consistency of genotype frequencies with Hardy-Weinberg equilibrium (HWE). We compared author names, authors’ affiliations, and geographic locations and period of studies to identify sequential or multiple publications. If more than one report related to the same or overlapping data sets, we included results from the largest or most recent publication.

### Statistical Analysis

We calculated odds ratios with 95% confidence intervals (CIs) to assess the strength of the association between the *IL10* gene polymorphisms and SLE risk. For each allele or haplotype with enough data sets, we performed overall analysis as well as subgroup analysis on the basis of population ethnicity and HWE in control groups, as genotype frequencies are often different across ethnicities and deviating from HWE may be a sign of selection bias or population stratification. HWE was tested using the χ^2^ test and it was considered statistically significant when the *P* value is less than 0.05 [22].

For SNPs, we adopt four genetic models to evaluate their association with SLE risk: major allele vs. minor allele, major allele homozygotes vs. minor allele homozygotes, major allele homozygotes vs. heterozygotes plus minor allele homozygotes, and major allele homozygotes plus heterozygotes vs. minor allele homozygotes.

Heterogeneity was determined using the *P* value from the χ^2^ test (Cochran’s *Q* statistic) and the *I* ^2^ statistic. The *I* ^2^ statistic represents the proportion of variation in the study estimates due to heterogeneity, in which 0–40% may be unimportant heterogeneity, 30–60% indicates moderate, 50–90% indicates substantial and 75–100% indicates considerable heterogeneity [23]. When the *P* value from the χ^2^ test was more than 0.10, the summary OR estimate was calculated by the fixed-effect model [24]. Otherwise, the random-effect model was used [25]. Publication bias was investigated by funnel plot and Egger’s linear regression test [26]. All statistical analyses were done with STATA version 10.0 (StataCorp LP, College Station, Texas, USA). This meta-analysis does not have a protocol. The PRISMA (Preferred Reporting Items for Systematic Reviews and Meta-Analyses) checklist is available in supplementary materials (see Method S2).

## Results

### Study Characteristics

**Figure 1:**
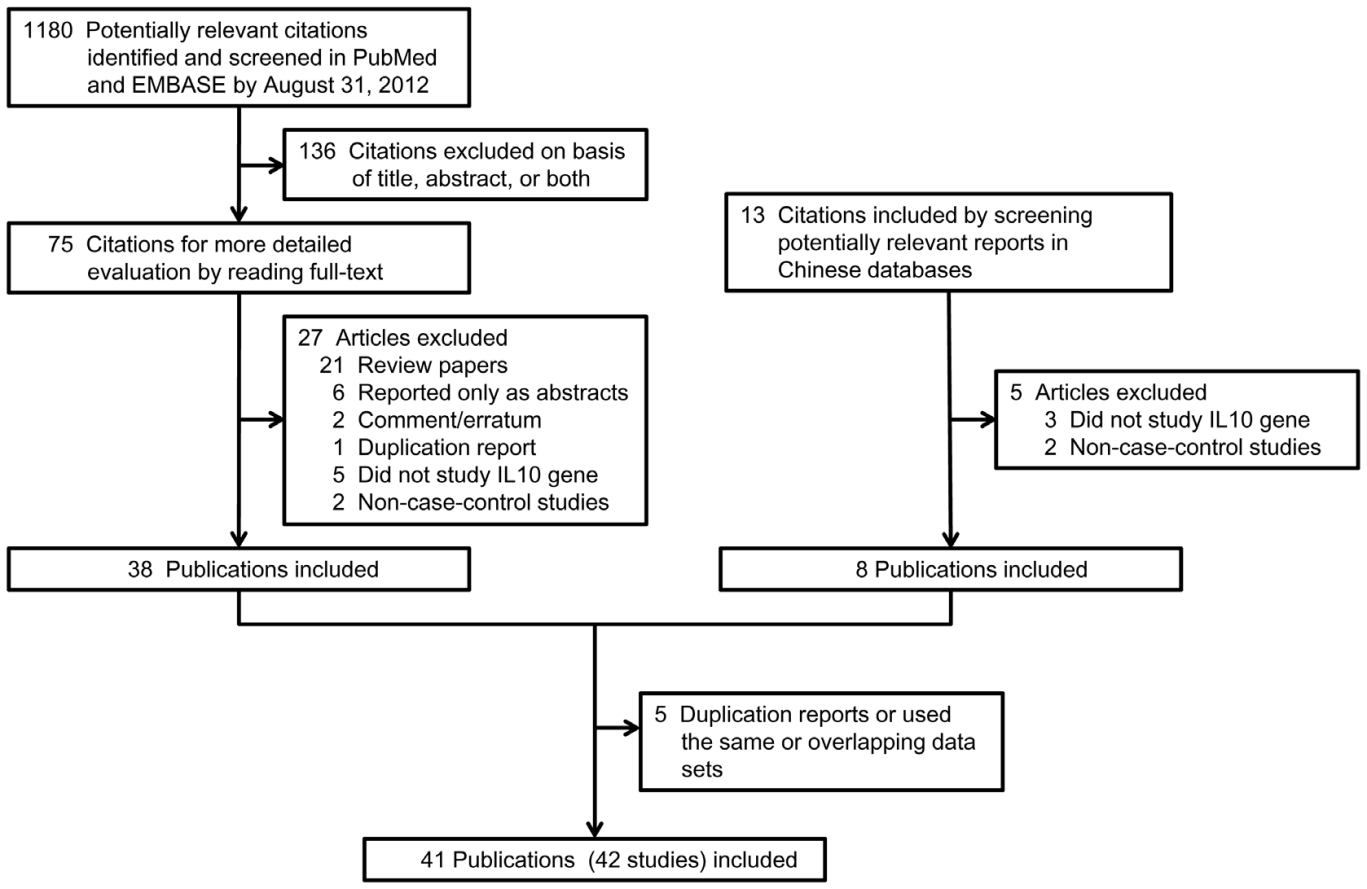
Flow diagram of the study selection process.

By screening title or abstract and further evaluating full-text, we identified 38 case-control studies from PubMed and EMBASE and 8 from Chinese Biomedical Literature Database. 5 of them were excluded because of duplication reports or using the same or overlapping data sets. One publication contained two individual case-control studies. At last, 41 publications with 42 case-control studies were included in this review (see Reference S1). Figure 1 describes the study selection process.

These 42 case-control studies were published from 1997 to 2011 with 33 in English, 6 in Chinese, 1 in Russian and 1 in Bulgarian. They included 7948 cases and 11866 controls. All studies used healthy people as controls. Ethnic groups among these studies were as following: 20 were Asians, 16 were Caucasians, 2 were Mexicans, 1 was African, 1 was Colombian, 1 was Kazakh, and 1 was mixed populations. Table 1 shows a brief description of these 42 case-control studies.

**Table 1:** Characteristics of included studies in this meta-analysis.

Study	Ref.	Ethnicity	Country	SLE	Control	Genotype method	Studied polymorphisms	Findings
Eskdale (1997)	1	Caucasian	UK	56	102	Genescan	IL10.G, IL10.R	CA21, CA25 associated with SLE
Lazarus (1997)	2	Caucasian	UK	76	119	ASO	−1082, −819, −592	No association
Mehrian (1998)	3	Mexican	USA	158	220	Genescan	IL10.G	CA22, CA23 associated with SLE (P = 0.0001, P = 0.015, respectively)
Mok (1998)	4	Asian	China	88	83	RFLP	−1082, −819, −592	No association
Ou (1998)	5	Asian	China	100	103	Genescan	IL10.G	No association
Alarcon-Riquelme (1999)	6	Mexican	Mexico	330	368	Genescan	IL10.G	No association
Crawley (1999)	7	Caucasian	UK	120	274	ASO	−1082, −819, −592	No association
Rood (1999)	8	Caucasian	Netherlands	92	162	ASO	−1082, −819, −592	No association
D'Alfonso 1 (2000)	9	Caucasian	Italy	98	104	Genescan	IL10.G, IL10.R	CA23 associated with SLE (P = 0.0425)
D'Alfonso 2 (2000)	10	Caucasian	Italy	68	64	Genescan	IL10.G	CA23 associated with SLE (P = 0.0470)
Gibson (2002)	11	African	USA	60	64	Sequencing	−3575, −2849, −2763	−2763 associated with SLE (P<0.05)
D'Alfonso (2002)	12	Caucasian	Italy	205	631	HPLC	26 SNPs including −1082, −592, −3575,−2849#, −2763 and IL10.G	IL10G long alleles (CA repeat >21) associated with SLE
Dijstelbloem (2002)	13	Caucasian	Netherlands	180	163	ASO	−1082	No association
Guseva (2003)	14	Kazakh	Russia	49	71	PCR-SSP	−592	−592 associated with SLE (P = 0.003)
Shen (2003)	15	Asian	China	220	230	Genescan, PCR-SSP	IL10G, IL10R, −1082, −819, −592	CA21, CA22, −1082 associated with SLE (P = 0.009, 0.006 and <0.0001, respectively)
Chong (2004)	16	Asian	China	554	708	Genescan, Taqman,RFLP	IL10.G, IL10.R #, −1082, −819, −592, −3575,−2849 #, −2763	−592, CA16 associated with SLE (0.009 and 0.017, respectively)
Fei (2004)	17	Caucasian	Sweden	52	26	RFLP	−1082	−1082 associated with SLE (P<0.05)
Schotte (2004)	18	Caucasian	Germany	210	158	Genescan	IL10.G, IL10.R	No association
Guzowski (2005)	19	Mixed	USA	48	25	HPLC	−1082, −819, −592	Not estimate
Hrycek (2005)	20	Caucasian	Poland	24	36	PCR-SSP	−1082, −819, −592	No association
Khoa (2005)	21	Asian	Vietnam	64	57	PCR-SSP	−1082	−1082 associated with SLE (P<0.05)
Lu (2005)	22	Asian	China	136	115	RFLP	−819	−819 associated with SLE (P = 0.009)
Suarez (2005)	23	Caucasian	Spain	187	343	ASO	−1082, −592	No association
Zhu (2005)	24	Asian	China	265	100	RFLP	−592	No association
Chen (2006)	25	Asian	China	237	304	Genescan	IL10.G	CA20 associated with SLE (P<0.0001)
Hirankarn (2006)	26	Asian	Thailand	195	159	MS	−1082, −819, −592	Haplotype ACC associated with SLE (P = 0.03)
Sung (2006)	27	Asian	Korea	350	330	SNaPshot	9 SNPs including −1082, −819, −592	No association
Guarnizo-Zuccardi (2007)	28	Colombian	Colombia	120	102	PCR-SSP	−1082, −819, −592	No association
Lin (2007)	29	Asian	China	119	100	RFLP	−592	−592 associated with SLE (P = 0.003)
Lan (2007)	30	Asian	China	90	110	RFLP	−1082, −819, −592	−1082 and haplotype GCC associated with SLE (P<0.05)
Wang (2007)	31	Asian	China	83	125	RFLP	−1082, −819, −592	−819, −592, haplotype ACC, haplotype ATA associated with SLE (P<0.001)
Xu (2007)	32	Asian	China	103	110	RFLP	−3575, −2763	−3575 associated with SLE (P<0.05)
Zhou (2007)	33	Asian	China	137	122	RFLP	−1082, −819, −592	−1082 and associated with SLE (P<0.001)
Hee (2008)	34	Asian	Malaysia	44	44	RFLP	−1082, −819, −592	haplotype GCC associated with SLE (P = 0.004)
Miyagawa (2008)	35	Asian	Japan	264	695	PLACE-SSCP	5 SNPs including −819, −592	No association
Rosado (2008)	36	Caucasian	Spain	116	151	Genescan, RFLP	IL10G, IL10R, −1082, −819, −592	Haplotype GCC, ACC (P<0.05)
Gateva (2009)	37	Caucasian	USA, Sweden	1963	4329	SNP array	Over 12000 variants	rs3024505 (P = 3.95×10^−8^)
Sobkowiak (2009)	38	Caucasian	Poland	103	300	Sequencing	−1082, −819, −592	Haplotype GCC (P = 0.0022)
Lin (2010)	39	Asian	China	172	215	Taqman	−1082, −819, −592	Haplotype ATA (P<0.001)
Miteva (2010)	40	Caucasian	Bulgaria	157	126	PCR-SSP	−1082	No association
Yu (2010)	41	Asian	China	110	138	SNPstream	−1082, −819, −592	No association
Ren (2011)	42	Asian	China	145	80	RFLP	−592	No association

ASO: allele-specific oligonucleotid hybridization; RFLP: restriction fragment length polymorphism; PCR-SSP: polymerase chain reaction sequence specific primer; HPLC: high-performance liquid chromatography; MS: mass spectrometry; PLACE-SSCP post-PCR fluorescent labeling and automated capillary electrophoresis under single-strand conformation polymorphism conditions; SNPs: single nucleotide polymorphisms.

# no polymorphisms.

Of the 42 studies investigating the association of the *IL-10* gene polymorphisms with SLE, 11 studied the *IL10.G* microsatellites, 6 studied the *IL-10.R* microsatellites, 24 studied −1082, 20 studied −819, 26 studied −592, 4 studied −3575 and 4 studied −2763. For other SNPs, there was only one study.

### Association of the IL10.G and IL10.R Microsatellites with SLE Susceptibility

Of the 11 studies investigating the association between the *IL10.*G microsatellites and SLE susceptibility, 10 provided enough data to calculate ORs (Table 2). By pooling the 10 studies, the meta-analyses showed that only CA27 allele of the *IL10.G* was associated with SLE (OR 1.32, 95% CI 1.01–1.72) and there was no significant between-study heterogeneity. Further subgroup analyses showed that this association was found among Caucasians (OR 2.38, 95% CI 1.01–5.62), but not among other populations. Subgroup analyses also showed that the CA21 allele of the *IL10.G* was associated with SLE among Asians (OR 1.28, 95% CI 1.02–1.60). But there was substantial between-study heterogeneity. As described previously, we also divided all alleles of the *IL10.*G microsatellites into long allele (>21 CA repeats) and short allele (≤21 CA repeats). No association was found between them and SLE.

**Table 2:** Meta-analysis of the IL-10.G and IL-10.R microsatellites with SLE.

Genetic model	Population	Study	OR (95% CI)	Statistical model	I^2^	*P*_Heterogeneity_	*P*_Egger’s test_
IL-10.G CA21	Overall	10	1.03 (0.85–1.25)	Random	77.0%	<0.001	0.512
	Caucasian	4	0.86 (0.62–1.20)	Random	58.9%	0.063	0.327
	Mexican	2	0.84 (0.42–1.76)	Random	92.9%	<0.001	–
	Asian	4	**1.28 (1.02–1.60)**	Random	67.4%	0.027	0.199
IL-10.G CA22	Overall	10	1.10 (0.83–1.45)	Random	72.1%	<0.001	0.352
	Caucasian	4	1.33 (0.88–1.99)	Fixed	0.0%	0.947	0.170
	Mexican	2	1.59 (0.54–4,69)	Random	94.2%	<0.001	–
	Asian	4	0.86 (0.73–1.01)	Fixed	45.8%	0.137	0.829
IL-10.G CA23	Overall	10	1.18 (0.88–1.57)	Random	41.5%	0.081	0.555
	Caucasian	4	1.30 (0.76–2.25)	Random	61.8%	0.049	0.770
	Mexican	2	1.22 (0.88–1.70)	Fixed	62.8%	0.101	–
	Asian	4	0.96 (0.61–1.49)	Fixed	25.2%	0.260	0.371
IL-10.G CA24	Overall	10	0.84 (0.68–1.03)	Fixed	5.9%	0.387	0.798
	Caucasian	4	0.93 (0.51–1.69)	Random	54.5%	0.086	0.484
	Mexican	2	0.87 (0.63–1.20)	Fixed	0.0%	0.452	–
	Asian	4	0.77 (0.54–1.11)	Fixed	0.0%	0.551	0.267
IL-10.G CA25	Overall	10	0.77 (0.58–1.03)	Random	77.2%	<0.001	0.490
	Caucasian	4	0.54 (0.27–1.05)	Random	87.8%	<0.001	0.577
	Mexican	2	1.00 (0.78–1.28)	Fixed	0.0%	0.646%	–
	Asian	4	0.92 (0.74–1.13)	Fixed	1.8%	0.383	0.710
IL-10.G CA26	Overall	10	1.04 (0.89–1.22)	Fixed	0.0%	0.816	0.369
	Caucasian	4	1.11 (0.77–1.61)	Fixed	0.0%	0.763	0.968
	Mexican	2	0.88 (0.62–1.23)	Fixed	0.0%	0.350	–
	Asian	4	1.08 (0.89–1.32)	Fixed	0.0%	0.582	0.147
IL-10.G CA27	Overall	10	**1.32 (1.01–1.72)**	Fixed	8.2%	0.366	0.561
	Caucasian	4	**2.38 (1.01–5.62)**	Fixed	0.0%	0.785	0.536
	Mexican	2	1.22 (0.83–1.79)	Fixed	56.5%	0.129	–
	Asian	4	1.23 (0.82–1.86)	Fixed	35.0%	0.201	0.213
IL-10.G CA28	Overall	9	1.06 (0.72–1.58)	Fixed	0.5%	0.429	0.839
	Caucasian	3	1.03 (0.25–4.22)	Fixed	0.0%	0.699	–
	Mexican	2	0.67 (0.28–1.57)	Fixed	0.0%	0.542	–
	Asian	4	1.24 (0.77–2.01)	Fixed	46.8%	0.130	0.946
IL-10.G Long allele	Overall	10	0.85 (0.64–1.12)	Random	88.5%	<0.001	0.300
	Caucasian	4	0.64 (0.28–1.45)	Random	93.1%	<0.001	0.392
	Mexican	2	1.25 (0.57–2.75)	Random	94.7%	<0.001	–
	Asian	4	0.86 (0.68–1.10)	Random	70.3%	0.018	0.322
IL-10.R R2	Caucasian	3	0.97 (0.77–1.23)	Random	63.9%	0.063	0.415
IL-10.R R3	Caucasian	3	1.00 (0.67–1.50)	Random	60.9%	0.077	0.308
IL-10.R R4	Caucasian	3	0.85 (0.38–1.89)	Fixed	0.0%	0.443	0.702

OR: odds ratio; CI: confidence interval; SLE: systemic lupus erythematosus.

For the *IL10.R* microsatellites, only 3 studies were available to calculate ORs. They all studied Caucasians. The meta-analyses found no association between the *IL10.R* microsatellites and SLE.

### Association of the IL10 −1082G/A Polymorphism with SLE Susceptibility

Of the 24 studies investigating the association between the *IL10* −1082G/A polymorphism and SLE susceptibility, 23 provided enough data to calculate ORs (Table 3). The results of pooling all studies showed that the *IL10* −1082 G/A polymorphism was not associated with SLE susceptibility under any genetic models. After excluding studies deviating from HWE [27], [28], the results showed that the *IL10* −1082G allele was associated with increased SLE risk under three genetic models (G vs. A: OR 1.21, 95% CI 1.02–1.44; GG vs. AA: OR 1.45, 95% CI 1.16–1.82; GG+GA vs. AA: OR 1.16, 95% CI 1.03–1.29).

**Table 3:** Meta-analysis of the *IL10* −1082G/A polymorphism and SLE.

Genetic model	Population	Study	OR (95% CI)	Statistical model	I^2^	*P*_Heterogeneity_	*P*_Egger’s test_
G vs. A	Overall	23	1.13 (0.92–1.37)	Random	78.5%	<0.001	0.628
		21*	**1.21 (1.02–1.44)**	Random	69.2%	<0.001	0.797
		20#	**1.15 (1.05–1.25)**	Fixed	26.4%	0.135	0.299
	Caucasian	10	**1.16 (1.04–1.28)**	Fixed	28.3%	0.184	0.885
		9*	**1.15 (1.03–1.28)**	Fixed	33.8%	0.147	0.996
	Asian	11	1.08 (0.65–1.81)	Random	88.6%	<0.001	0.558
		10*	1.28 (0.84–1.95)	Random	80.0%	<0.001	0.185
		9#	1.19 (0.98–1.44)	Fixed	32.1%	0.161	0.076
GG vs. AA	Overall	15	1.24 (0.82–1.86)	Random	66.9%	<0.001	0.608
		13*	**1.45 (1.16–1.82)**	Fixed	34.7%	0.104	0.892
	Caucasian	9	**1.43 (1.13–1.81)**	Fixed	33.5%	0.150	0.877
		8*	**1.40 (1.10–1.79)**	Fixed	40.4%	0.109	0.830
	Asian	4	0.99 (0.10–9.75)	Random	87.1%	<0.001	–
		3*	**3.21 (1.24–8.28)**	Fixed	18.4%	0.294	–
GG+GA vs. AA	Overall	17	1.07 (0.89–1.28)	Random	61.7%	<0.001	0.434
		15*	**1.16 (1.03–1.29)**	Fixed	0.0%	0.610	0.501
	Caucasian	9	**1.16 (1.03–1.32)**	Fixed	0.0%	0.749	0.982
		8*	**1.15 (1.01–1.31)**	Fixed	0.0%	0.706	0.838
	Asian	6	0.93 (0.51–1.69)	Random	84.7%	<0.001	0.948
		5*	1.26 (0.99–1.62)	Fixed	24.2%	0.260	0.560
GG vs. AA+GA	Overall	15	1.14 (0.81–1.61)	Random	66.1%	<0.001	0.804
		13*	1.30 (0.94–1.78)	Random	50.6%	0.019	0.907
	Caucasian	9	1.22 (0.89–1.67)	Random	56.1%	0.020	0.911
		8*	1.22 (0.85–1.76)	Random	61.5%	0.011	0.924
	Asian	4	1.18 (0.22–6.27)	Random	82.2%	0.001	–
		3*	**2.85 (1.19–6.79)**	Fixed	4.7%	0.350	–

OR: odds ratio; CI: confidence interval; SLE: systemic lupus erythematosus.

* exclude the studies deviating from Hardy-Weinberg equilibrium.

# exclude the study by Shen (2003).

In the subgroup analyses by ethnicity, the results showed that the *IL10* −1082G allele was associated with increased SLE risk among Caucasians under three genetic models (G vs. A: OR 1.16, 95% CI 1.04–1.28; GG vs. AA: OR 1.43, 95% CI 1.13–1.81; GG+GA vs. AA: OR 1.16, 95% CI 1.03–1.32), while the associations were not found among Asians under any genetic model. After excluding studies deviating from HWE [27], [28], the results hardly changed for Caucasians, while for Asians statistically significant associations were found under the genetic models of GG vs. AA (OR 3.21, 95% CI 1.24–8.28) and GG vs. AA+GA (OR 2.85, 95% CI 1.19–6.79) and a close but not statistically significant association was found under the genetic model of GG+GA vs. AA (OR 1.26, 95% CI 0.99–1.62).

The heterogeneity was significant in the pooling analyses of total available studies and in the subgroup analyses of Asians. Deviating from HWE in control groups can explain much of it. When excluding studies deviating from HWE could not eliminate the heterogeneity or eliminate it little, we excluded one more study. By this way, we found that the study by Shen contributed much heterogeneity in the pooling analysis and the subgroup analysis of Asians [29]. After excluding the study deviating from HWE and the study by Shen [28], [29], a close but not statistically significant association was found among Asians using the genetic model of G vs. A (OR 1.19, 95% CI 0.98–1.44). In the subgroup analyses of Caucasians, the heterogeneity was not important under genetic models of G vs. A, GG vs. AA and GG+GA vs. AA, but under the model of GG vs. AA+GA. Excluding studies deviating from HWE and excluding one more study eliminated the heterogeneity little. Results from funnel plot and Egger’s test suggested that publication bias was not evident (*P*_Egger’s test_>0.05, Table 3).

### Association of the IL10 −819C/T Polymorphism with SLE Susceptibility

Of the 20 studies investigating the association between the *IL10* −819C/T polymorphism and SLE susceptibility, 18 provided enough data to calculate ORs (Table 4). The results of pooling all studies showed that the *IL10* −819C/T polymorphism was not associated with SLE susceptibility under any genetic models. Ethnicity, consistency with HWE, and adjustment for heterogeneity did not affect the results.

**Table 4:** Meta-analysis of the *IL10* −819C/T polymorphism and SLE.

Genetic model	Population	Study	OR (95% CI)	Statistical model	I^2^	*P*	*P*_Egger’s test_
C vs. T	Overall	18	0.91 (0.75–1.10)	Random	73.5%	<0.001	0.232
		14*	0.90 (0.72–1.11)	Random	71.7%	<0.001	0.107
		13#	1.04 (0.93–1.17)	Fixed	11.5%	0.330	**0.002**
	Caucasian	6	0.95 (0.80–1.13)	Fixed	0.0%	0.700	0.138
	Asian	10	0.94 (0.69–1.28)	Random	84.6%	<0.001	0.580
		7*	0.91 (0.62–1.33)	Random	85.4%	<0.001	0.356
		6#	1.15(0.99–1.34)	Fixed	3.7%	0.393	0.142
CC vs. TT	Overall	13	0.87 (0.66–1.16)	Fixed	29.0%	0.154	**0.013**
		9*	0.93 (0.65–1.33)	Fixed	0.0%	0.734	0.165
	Caucasian	6	0.90 (0.59–1.37)	Fixed	0.0%	0.712	0.449
	Asian	5	1.09 (0.71–1.68)	Fixed	47.7%	0.105	**0.002**
		2*	1.27 (0.62–2.64)	Fixed	0.0%	0.482	–
CC+CT vs. TT	Overall	13	0.89 (0.63–1.25)	Random	59.0%	0.004	0.647
		9*	0.97 (0.72–1.31)	Fixed	0.0%	0.799	0.128
	Caucasian	6	0.91 (0.60–1.37)	Fixed	0.0%	0.712	0.629
	Asian	5	1.04 (0.59–1.81)	Random	81.1%	<0.001	0.569
		2*	1.12 (0.70–1.81)	Fixed	0.0%	0.398	–
CC vs. TT+CT	Overall	13	0.94 (0.79–1.13)	Fixed	0.0%	0.511	0.141
		9*	0.94 (0.77–1.15)	Fixed	0.0%	0.665	0.291
	Caucasian	6	0.94 (0.76–1.17)	Fixed	0.0%	0.679	0.146
	Asian	5	1.05 (0.69–1.59)	Fixed	27.0%	0.241	**0.014**
		2*	1.23 (0.63–2.42)	Fixed	0.0%	0.657	–

OR: odds ratio; CI: confidence interval; SLE: systemic lupus erythematosus.

* exclude the studies deviating from Hardy-Weinberg equilibrium.

# exclude the study by Wang (2007).

The heterogeneity was significant in the overall analyses and the subgroup analyses of Asians under the genetic models of C vs. T and CC+CT vs. TT. After excluding studies deviating from HWE and the study by Wang [28], [30], [31], [32], the heterogeneity was eliminated. Results from funnel plot and Egger’s test suggested that publication bias was present in the studies investigating the association between the *IL10* −819C/T polymorphism and SLE (Table 4).

### Association of the IL10 −592C/A Polymorphism with SLE Susceptibility

Of the 26 studies investigating the association between the *IL10* −592C/A polymorphism and SLE susceptibility, 24 provided enough data to calculate ORs (Table 5). By pooling all studies, the *IL10* −592 C allele was associated with decreased SLE risk under the genetic model of CC+CA vs. AA (OR 0.79, 95% CI 0.64–0.99). A close but not statistically significant association was found under the genetic model of C vs. A (OR 0.87, 95% CI 0.74–1.01). After excluding studies deviating from HWE [28], [33], [34], statistically significant associations were found under the genetic models of C vs. A (OR 0.84, 95% CI 0.70–1.00), CC vs. AA (OR 0.66, 95% CI 0.44–0.99) and CC+CA vs. AA (OR 0.73, 95% CI 0.62–0.88), and a close but not statistically significant association was found under the genetic model of CC vs. AA+CA (OR 0.77, 95% CI 0.59–1.01).

**Table 5:** Meta-analysis of the *IL10* −592C/A polymorphism and SLE.

Genetic model	Population	Study	OR (95% CI)	Statistical model	I^2^	*P*_Heterogeneity_	*P*_Egger’s test_
C vs. A	Overall	24	0.87 (0.74–1.01)	Random	76.0%	<0.001	**0.021**
		21*	**0.84 (0.70–1.00)**	Random	76.4%	<0.001	0.104
	Caucasian	8	1.02 (0.89–1.16)	Fixed	5.6%	0.387	0.247
	Asian	13	0.85 (0.67–1.09)	Random	84.5%	<0.001	0.120
		11*	0.80 (0.60–1.07)	Random	84.5%	<0.001	0.407
CC vs. AA	Overall	18	0.72 (0.48–1.07)	Random	69.1%	<0.001	**0.008**
		15*	**0.66 (0.44–0.99)**	Random	59.6%	0.002	0.221
	Caucasian	7	1.08 (0.75–1.55)	Fixed	0.0%	0.483	0.223
	Asian	8	0.71 (0.36–1.39)	Random	81.6%	<0.001	0.068
		6*	0.51 (0.24–1.06)	Random	72.5%	0.003	0.599
CC+CA vs. AA	Overall	18	**0.79 (0.64–0.99)**	Random	50.4%	0.008	0.194
		15*	**0.73 (0.62–0.88)**	Fixed	32.5%	0.109	0.425
	Caucasian	7	1.06 (0.74–1.51)	Fixed	0.0%	0.575	0.330
	Asian	8	0.80 (0.60–1.06)	Random	67.2%	0.003	0.292
		6*	**0.69 (0.51–0.94)**	Random	49.6%	0.078	0.309
CC vs. AA+CA	Overall	18	0.86 (0.67–1.10)	Random	63.6%	<0.001	**0.003**
		15*	0.77 (0.59–1.01)	Random	59.6%	0.002	**0.004**
	Caucasian	7	1.05 (0.87–1.26)	Fixed	2.1%	0.409	0.051
	Asian	8	0.79 (0.44–1.43)	Random	78.0%	<0.001	0.054
		6*	0.59 (0.31–1.12)	Random	67.5%	0.009	0.435

OR: odds ratio; CI: confidence interval; SLE: systemic lupus erythematosus.

* exclude the studies deviating from Hardy-Weinberg equilibrium.

In the subgroup analyses by ethnicity, the results showed that only under the genetic model of CC+CA vs. AA the *IL10* −592 C allele was associated with decreased SLE risk among Asians (OR 0.69, 95% CI 0.51–0.94). The associations were not found among Caucasians under any genetic model.

The heterogeneity was significant in the pooling analyses of total available studies and in the subgroup analyses of Asians, and was still significant after excluding studies deviating from HWE [28], [33]. Results from funnel plot and Egger’s test suggested that publication bias was present in the studies investigating the association between the *IL10* −592C/A polymorphism and SLE susceptibility (Table 5).

### Association of the IL10 −1082/−819/−592 Haplotype with SLE Susceptibility

There were 16 studies investigating the association between the IL10 −1082/−819/−592 haplotype and SLE susceptibility. GCC, ACC and ATA were the only three haplotypes or account for the vast majority of the IL10 −1082/−819/−592 haplotypes in these studies. Therefore, we just evaluated the three haplotypes (Table 6). The overall meta-analyses showed that the GCC (OR 1.21, 95% CI 1.05–1.40) and the ACC (OR 0.75, 95% CI 0.60–0.95) haplotypes were associated with SLE risk. Further subgroup analyses showed that the GCC haplotype was associated with increased SLE risk among Caucasians (OR 1.25, 95% CI 1.10–1.42) and Asians (OR 1.24, 95% CI 1.00–1.53)and the ACC haplotype was associated with decreased SLE risk among Caucasians (OR 0.77, 95% CI 0.61–0.97) but not among Asians (OR 0.74, 95% CI 0.50–1.12). Excluding the studies deviating from HWE [27], [28], [30], [33] yielded similar results except in the subgroup analysis of Asians for the GCC haplotype (OR 1.01, 95% CI 0.75–1.36) and Caucasians for the ACC haplotype (OR 0.79, 95% CI 0.62–1.02).

**Table 6:** Meta-analysis of the *IL10* −1082/−819/−592 haplotype and SLE.

Genetic model	Population	Study	OR (95% CI)	Statistical model	I^2^	*P*_Heterogeneity_	*P*_Egger’s test_
GCC vs. others	Overall	16	**1.21 (1.05–1.40)**	Random	33.0%	0.098	0.407
		12*	**1.18 (1.04–1.33)**	Fixed	36.0%	0.103	0.076
	Caucasian	7	**1.25 (1.10–1.42)**	Fixed	40.5%	0.121	0.876
		6*	**1.24 (1.01–1.51)**	Random	49.9%	0.076	0.839
	Asian	8	**1.24(1.00–1.53)**	Fixed	29.3%	0.194	0.600
		5*	1.01 (0.75–1.36)	Fixed	0.0%	0.450	0.124
ACC vs. others	Overall	16	**0.75 (0.60–0.95)**	Random	82.7%	<0.001	0.052
		12*	**0.73 (0.53–0.99)**	Random	84.4%	<0.001	0.350
	Caucasian	7	**0.77 (0.61–0.97)**	Random	56.2%	0.033	0.207
		6*	0.79 (0.62–1.02)	Random	59.1%	0.032	0.333
		5#	0.91 (0.77–1.07)	Fixed	0.0%	0.446	0.206
	Asian	8	0.74 (0.50–1.12)	Random	90.2%	<0.001	0.191
		5*	0.66 (0.31–1.40)	Random	93.0%	<0.001	0.649
ATA vs. others	Overall	16	1.14 (0.94–1.39)	Random	77.7%	<0.001	0.073
		12*	1.21 (0.91–1.60)	Random	81.1%	<0.001	0.331
	Caucasian	7	0.96 (0.82–1.11)	Fixed	19.2%	0.284	0.058
		6*	0.94 (0.80–1.10)	Fixed	28.3%	0.223	0.108
	Asian	8	1.22 (0.87–1.71)	Random	87.7%	<0.001	0.195
		5*	1.42 (0.80–2.52)	Random	89.8%	<0.001	0.874

OR: odds ratio; CI: confidence interval; SLE: systemic lupus erythematosus.

* exclude the studies deviating from Hardy-Weinberg equilibrium.

# exclude the study by Sobkowiak (2009).

The heterogeneity was significant in the overall analyses. Some of the heterogeneity can be resolved by ethnicity-specific analyses. However, the heterogeneity remained in some ethnicity-specific analyses (ACC among Caucasians and Asians, and ATA among Asians). Excluding the study by Sobkowiak [35] eliminated much of the heterogeneity among Caucasian studies, while for Asians the heterogeneity had no significant change after excluding one study. Funnel plot and Egger’s test suggested that publication bias was not apparent (Table 6).

### Association of other SNPs in the IL10 Gene with SLE Susceptibility

There were four studies investigating the association between the *IL10* −3575T/A polymorphism and SLE susceptibility. The meta-analyses showed no association under any genetic model. The *I* ^2^ statistic showed that the between-study heterogeneity was not apparent.

There were three studies investigating the association between the *IL10* −2763C/A polymorphism and SLE susceptibility. The meta-analyses showed that the *IL10* −2763 C allele was associated with increased SLE risk (CC+CA vs. AA: OR 2.64, 95% CI 1.01–6.84, fixed model, *I* ^2^ = 57.8%, *P*_Heterogeneity_ = 0.124). Further analysis showed that the study by Gibson contributed to this association. In this study, the authors studied African Americans.

For other SNPs in the *IL10* gene, only rs3024505 was associated with SLE among Caucasians (1 study, *P* = 3.95×10^−8^).

## Discussion

IL-10 is a potent stimulator of B cells in one way and a strong inhibitor of antigen-presenting cells and T cells in another way. Therefore, it plays an important role in immune and inflammatory process and aberrant expression of IL-10 contributes to the development of autoimmune diseases [36]. Polymorphisms in the IL-10 gene may alter IL-10 production, and thus influencing susceptibility to autoimmune diseases. For asthma, rheumatoid arthritis, type 1 diabetes, and graft-versus-host disease, observational studies have demonstrated the gene-disease association [20]. In the past 15 years, a number of case-control studies have also been conducted to investigate the association in SLE. In this meta-analysis, we collected all available published case-control studies on the association between the IL-10 gene polymorphisms and SLE susceptibility and combined them when combinable, hoping to give a whole picture of this topic.

Previous meta-analysis has found that the CA23 allele of the *IL-10.G* microsatellites is associated with SLE [37]. Our meta-analysis, which included more studies, failed to replicate this finding. But our meta-analysis showed an association between the CA27 allele and SLE in Caucasian patients without between-study heterogeneity. This association has never been reported in each individual study. Thus, our meta-analysis produced a new finding about the association between the *IL-10.G* microsatellites and SLE. In addition, we found an association between the CA21 allele and SLE in Asian patients with between-study heterogeneity. However, it should be noticed that different alleles in the *IL-10.G* microsatellites have been linked with SLE risk in different studies. Ethnicity and patient heterogeneity may explain it. The allele distributions of the *IL-10.G* microsatellites are often different among different populations; some subgroup patients such as patients with anti-Sm antibodies or with nephritis tend to correlate with certain *IL-10.G* alleles, while their distributions are different among different studies [38], [39]. It is also likely that the *IL-10.G* microsatellites are markers of disease susceptibility due to linkage disequilibrium with some causal variation.

This meta-analysis showed a relative solid association between the *IL-10* −1082 G/A polymorphisms and SLE among Caucasians, because the results were consistent under three genetic models (G vs. A, GG vs. AA, and GG+GA vs. AA) without between-study heterogeneity and insensitive to HWE status in control groups. An earlier meta-analysis, because of very limited studies included, failed to result in a positive finding [37]. Two later meta-analyses, which included more studies but still fewer than ours, yielded similar findings with ours [40], [41]. For Asians, studies deviating from HWE and the study by Shen [29]were the main source of heterogeneity in this meta-analysis. Excluding these studies resulted in a significant association under two genetic models and a close but not significant association under the other models. Previous meta-analyses also supported an association of the *IL-10* −1082 G/A polymorphisms among Asians, but the numbers of included Asian studies were smaller than ours [37], [41]. Turner et al. reported that the *IL-10* −1082G allele was associated with higher IL-10 production following ConA stimulation of human peripheral blood lymphocytes *in vitro* [42]. Other researchers performed similar assays and reported contradictory associations between IL-10 production and genotype [43]. Therefore, functional studies do not fully support the hypothesis that the *IL-10* −1082 G/A polymorphisms have an effect on IL-10 production and thus influencing disease susceptibility. The IL-10 −1082 G/A polymorphisms may also be a marker of disease susceptibility due to linkage disequilibrium.

All studies from Europe showed a complete linkage disequilibrium at positions −819 and −592 in the *IL-10* gene. Thus, among Caucasians our meta-analysis produced consistent findings for the *IL-10* −819 C/T and −592 C/A. But for studies from Asia, 4 of them did not show a complete linkage disequilibrium at the two positions [29], [30], [44], [45]. Therefore, among Asians our meta-analysis produced inconsistent findings. After excluding studies deviating from HWE, we found that among Asians the *IL-10* −592 C/A polymorphisms were significantly associated with SLE under the genetic model of CC+CA vs. AA and showed a trend of association but not statistically significant under other models. This may contribute to positive findings from overall analyses of the IL-10 −592 C/A polymorphisms when excluding studies deviating from HWE. Eskdale et al. reported that the C allele at −592 of the *IL-10* gene or its associated haplotype was related to increased production of IL-10 by whole blood or PBMCs [46], while other researchers reported conflicting associations [47]. For patients with different diseases, the direction of association was also different between IL-10 production and the *IL-10* −592 C/A polymorphisms [48], [49], [50]. Therefore, the evidence from functional studies is not strong enough to support a causal association between them.

Because of linkage disequilibrium, GCC, ACC and ATA were the only three haplotypes or account for the vast majority of the *IL10* −1082/−819/−592 haplotypes in included studies. Overall meta-analysis showed that the GCC haplotype was associated with increased and the ACC haplotype with decreased SLE susceptibility and the results were sensitive to ethnicity and HWE status in control groups. These findings were similar with a recent meta-analysis [40]. For the ATA haplotype, our meta-analysis did not produce any association with SLE, which is conflicting with the recent meta-analysis [40]. Considering the absolutely more studies included in our meta-analysis, we are more confident with our findings. As the GCC haplotype was almost the only source of G allele at −1082 of the *IL-10* gene, similar findings should be yielded from the analysis of the GCC haplotype and the *IL-10* −1082 G/A polymorphisms. This can be seen in our present meta-analysis. Because of the almost complete linkage disequilibrium, functional findings about the *IL-10* −1082 G/A polymorphisms are suitable for the GCC haplotype.

This meta-analysis involving 7948 cases and 11866 controls used an exhaustive search strategy in recommended databases without language restrictions [51]. The number of included studies was at least twice as many as those in previous meta-analysis. Thus, our meta-analysis has more statistical power to detect positive findings and allow ethnicity-specific analysis. Our meta-analysis also identified and excluded studies that used overlapping data [52], [53], while previous meta-analyses treated them as separate study [37], [40], [41]. In addition, we performed sensitive analysis by excluding studies deviating from HWE. Some studies suggested that deviations from HWE in healthy populations may be a sign of selection bias or population stratification [54]. Consistent findings are solider from sensitive analysis by HWE.

Some caveats need to be noted regarding the present study. First, there was significant heterogeneity among included studies, especially among studies from Asia. Pan et al. reported that Chinese gene-disease association studies often have more heterogeneity than others [55]. In our systematic review and meta-analysis, most Asian studies were from China and some of them were the main source of heterogeneity. However, much of the heterogeneity can be eliminated through sensitive analysis. Second, publication bias from Egger’s test was apparent in some analyses of the *IL-10* −819 C/T and −592 C/A polymorphisms. This may be due to reporting bias, other biases or genuine heterogeneity, and it may be difficult to determine which is the case [51]. As we included more studies than previous meta-analyses and these meta-analyses did not show significant publication bias from Egger’s test, this is more likely to be due to other biases or genuine heterogeneity. Third, for the *IL10* −3575T/A and −2763C/A, relevant studies are quite few; there were also very limited studies conducted among black people; more studies needed to duplicate the association of rs3024505 with SLE. Fourth, to our knowledge, the SNPs that are related with SLE in this study have not been highlighted in recent GWAS focused on SLE except the SNP rs3024505. It is common that findings are different from candidate gene studies and GWAS studies. The reasons are currently hard to explain. Some possible reasons include: variation in demographic profile of controls, linkage disequilibrium in different populations, variation in accuracy of genotyping methods, false discovery, and confounding factors.

In summary, this meta-analysis showed that the CA27 allele of the *IL-10.G* microsatellites, the *IL-10* −1082G/A polymorphism and its associated haplotype of GCC are associated with SLE susceptibility among Caucasians and the CA21 allele of the *IL-10.G* microsatellites, the *IL-10* −1082G/A and −592 C/A polymorphisms and their associated haplotype of GCC may be associated with SLE susceptibility among Asians. It is worthwhile to note that this is the first time to report an association between the CA27 allele of the *IL-10.G* microsatellites and SLE among Caucasians. Further studies are needed to confirm these findings. More studies are also needed to duplicate the associations between SLE and some rarely studied SNPs such as rs3024505.

## Supporting Information

Methods S1 **Search strategies.**
(DOCX)

Methods S2 **Checklist to confirm compliance with PRISMA guidelines for systematic reviews and meta-analyses.**
(DOCX)

Reference S1 **List of included studies in this meta-analysis.**
(DOCX)
