# Supplementary material for: IL-10 Gene Polymorphisms and Susceptibility to Systemic Lupus Erythematosus: A Meta-Analysis
Source: PLoS One. 2013 Jul 23;8(7):e69547. doi: 10.1371/journal.pone.0069547 (PMC3720721; doi:10.1371/journal.pone.0069547)
Supplement: Methods S1 — Search strategies. (DOCX) [file pone.0069547.s001.docx]

**PubMed**

#1 Lupus erythematosus, systemic/

#2 systemic lupus erythematosus.tw.

#3 sle.tw.

#4 #1 or #2 or #3

#5 interleukin-10/

#6 interleukin-10.tw.

#7 il-10.tw.

#8 il10.tw.

#9 #5 or #6 or #7 or #8

#10 #4 and #9

**EMBASE**

#1 Systemic Lupus Erythematosus/
#2 systemic lupus erythematosus.ti.ab.
#3 sle.ti.ab.

#4 #1 or #2 or #3

#5 interleukin-10/

#6 interleukin-10.ti.ab.

#7 il-10.ti.ab.

#8 il10.ti.ab.

#9 #5 or #6 or #7 or #8

#10 #4 and #9

**CBM**

#1 缺省[智能]:系统性红斑狼疮

#2缺省[智能]: “Systemic Lupus Erythematosus”

#3缺省[智能]:SLE

#4 #1 or #2 or #3

#5缺省[智能]:白介素10

#6缺省[智能]:白介素-10

#7缺省[智能]:白介10

#8缺省[智能]:白介-10

#9缺省[智能]: “interleukin 10”

#10缺省[智能]:il-10

#11缺省[智能]: il10

#12 #5 or #6 or #7 or #8 or #9 or #10 or #11

#13 #4 and #12

**WanFang (theses and dissertations)**

(系统性红斑狼疮 OR SLE) AND (il10 OR il-10 OR 白介素-10 OR 白介素10) AND (基因 OR 多态性)

**CNKI (theses and dissertations)**

Abstract: (SLE OR系统性红斑狼疮) AND (il10 OR il-10) AND (基因 OR 多态性)
