# Supplementary material for: IL-10 Gene Polymorphisms and Susceptibility to Systemic Lupus Erythematosus: A Meta-Analysis
Source: PLoS One. 2013 Jul 23;8(7):e69547. doi: 10.1371/journal.pone.0069547 (PMC3720721; doi:10.1371/journal.pone.0069547)
Supplement: Reference S1 — List of included studies in this meta-analysis. (DOCX) [file pone.0069547.s003.docx]

**Reference S1. List of included studies in this meta-analysis**

1. Miteva L, Manolova I, Ivanova M, Stoilov R, Rashkov R, et al. (2010) Lack of association between promoter polymorphism -1082A/G in interleukin-10 gene and genetic predisposition to systemic lupus erythematosus. Revmatologia 18: 33-38.

2. Alarcon-Riquelme ME, Lindqvist AK, Jonasson I, Johanneson B, Sandino S, et al. (1999) Genetic analysis of the contribution of IL10 to systemic lupus erythematosus. J Rheumatol 26: 2148-2152.

3. Chen JY, Wang CM, Lu SC, Chou YH, Luo SF (2006) Association of apoptosis-related microsatellite polymorphisms on chromosome 1q in Taiwanese systemic lupus erythematosus patients. Clin Exp Immunol 143: 281-287.

4. Chong WP, Ip WK, Wong WH, Lau CS, Chan TM, et al. (2004) Association of interleukin-10 promoter polymorphisms with systemic lupus erythematosus. Genes Immun 5: 484-492.

5. Crawley E, Woo P, Isenberg DA (1999) Single nucleotide polymorphic haplotypes of the interleukin-10 5' flanking region are not associated with renal disease or serology in Caucasian patients with systemic lupus erythematosus. Arthritis Rheum 42: 2017-2018.

6. D'Alfonso S, i M, Bocchio D, Colombo G, Scorza-Smeraldi R, et al. (2000) Systemic lupus erythematosus candidate genes in the Italian population: evidence for a significant association with interleukin-10. Arthritis Rheum 43: 120-128.

7. Dijstelbloem HM, Hepkema BG, Kallenberg CG, van der Linden MW, Keijsers V, et al. (2002) The R-H polymorphism of FCgamma receptor IIa as a risk factor for systemic lupus erythematosus is independent of single-nucleotide polymorphisms in the interleukin-10 gene promoter. Arthritis Rheum 46: 1125-1126.

8. Eskdale J, Wordsworth P, Bowman S, Field M, Gallagher G (1997) Association between polymorphisms at the human IL-10 locus and systemic lupus erythematosus. Tissue Antigens 49: 635-639.

9. Fei GZ, Svenungsson E, Frostegard J, Padyukov L (2004) The A-1087IL-10 allele is associated with cardiovascular disease in SLE. Atherosclerosis 177: 409-414.

10. Gateva V, Sandling JK, Hom G, Taylor KE, Chung SA, et al. (2009) A large-scale replication study identifies TNIP1, PRDM1, JAZF1, UHRF1BP1 and IL10 as risk loci for systemic lupus erythematosus. Nat Genet 41: 1228-1233.

11. Gibson AW, Edberg JC, Wu J, Westendorp RG, Huizinga TW, et al. (2001) Novel single nucleotide polymorphisms in the distal IL-10 promoter affect IL-10 production and enhance the risk of systemic lupus erythematosus. J Immunol 166: 3915-3922.

12. Guarnizo-Zuccardi P, Lopez Y, Giraldo M, Garcia N, Rodriguez L, et al. (2007) Cytokine gene polymorphisms in Colombian patients with systemic lupus erythematosus. Tissue Antigens 70: 376-382.

13. Guseva IA, Omarbekova Zh E, Miakotkin VA (2003) [Polymorphism of Fc gamma RIIIA-158F/V gene and promoter region of IL-10 gene in systemic lupus erythematosus in Kazakhs]. Ter Arkh 75: 36-41.

14. Guzowski D, Chandrasekaran A, Gawel C, Palma J, Koenig J, et al. (2005) Analysis of single nucleotide polymorphisms in the promoter region of interleukin-10 by denaturing high-performance liquid chromatography. J Biomol Tech 16: 154-166.

15. Hee CS, Gun SC, Naidu R, Somnath SD, Radhakrishnan AK (2008) The relationship between single nucleotide polymorphisms of the interleukin-10 gene promoter in systemic lupus erythematosus patients in Malaysia: A pilot study. Int J Rheum Dis 11: 148-154.

16. Hirankarn N, Wongpiyabovorn J, Hanvivatvong O, Netsawang J, Akkasilpa S, et al. (2006) The synergistic effect of FC gamma receptor IIa and interleukin-10 genes on the risk to develop systemic lupus erythematosus in Thai population. Tissue Antigens 68: 399-406.

17. Hrycek A, Siekiera U, Cieslik P, Szkrobka W (2005) HLA-DRB1 and -DQB1 alleles and gene polymorphisms of selected cytokines in systemic lupus erythematosus. Rheumatol Int 26: 1-6.

18. Khoa PD, Sugiyama T, Yokochi T (2005) Polymorphism of interleukin-10 promoter and tumor necrosis factor receptor II in Vietnamese patients with systemic lupus erythematosus. Clin Rheumatol 24: 11-13.

19. Lazarus M, Hajeer AH, Turner D, Sinnott P, Worthington J, et al. (1997) Genetic variation in the interleukin 10 gene promoter and systemic lupus erythematosus. J Rheumatol 24: 2314-2317.

20. Lin PW, Huang CM, Huang CC, Tsai CH, Tsai JJ, et al. (2007) The association of -627 interleukin-10 promoter polymorphism in Chinese patients with systemic lupus erythematosus. Clin Rheumatol 26: 298-301.

21. Lin YJ, Wan L, Huang CM, Sheu JJ, Chen SY, et al. (2010) IL-10 and TNF-alpha promoter polymorphisms in susceptibility to systemic lupus erythematosus in Taiwan. Clin Exp Rheumatol 28: 318-324.

22. Lu LY, Cheng HH, Sung PK, Tai MH, Yeh JJ, et al. (2005) Tumor necrosis factor-beta +252A polymorphism is associated with systemic lupus erythematosus in Taiwan. J Formos Med Assoc 104: 563-570.

23. Mehrian R, Quismorio FP, Jr., Strassmann G, Stimmler MM, Horwitz DA, et al. (1998) Synergistic effect between IL-10 and bcl-2 genotypes in determining susceptibility to systemic lupus erythematosus. Arthritis Rheum 41: 596-602.

24. Miyagawa H, Yamai M, Sakaguchi D, Kiyohara C, Tsukamoto H, et al. (2008) Association of polymorphisms in complement component C3 gene with susceptibility to systemic lupus erythematosus. Rheumatology 47: 158-164.

25. Mok CC, Lanchbury JS, Chan DW, Lau CS (1998) Interleukin-10 promoter polymorphisms in Southern Chinese patients with systemic lupus erythematosus. Arthritis Rheum 41: 1090-1095.

26. Ou TT, Tsai WC, Chen CJ, Chang JG, Yen JH, et al. (1998) Genetic analysis of interleukin-10 promoter region in patients with systemic lupus erythematosus in Taiwan. Kaohsiung J Med Sci 14: 599-606.

27. Rood MJ, Keijsers V, van der Linden MW, Tong TQ, Borggreve SE, et al. (1999) Neuropsychiatric systemic lupus erythematosus is associated with imbalance in interleukin 10 promoter haplotypes. Ann Rheum Dis 58: 85-89.

28. Rosado S, Rua-Figueroa I, Vargas JA, Garcia-Laorden MI, Losada-Fernandez I, et al. (2008) Interleukin-10 promoter polymorphisms in patients with systemic lupus erythematosus from the Canary Islands. Int J Immunogenet 35: 235-242.

29. D'Alfonso S, Giordano M, Mellai M, Lanceni M, Barizzone N, et al. (2002) Association tests with systemic lupus erythematosus (SLE) of IL10 markers indicate a direct involvement of a CA repeat in the 5' regulatory region. Genes Immun 3: 454-463.

30. Schotte H, Gaubitz M, Willeke P, Tidow N, Assmann G, et al. (2004) Interleukin-10 promoter microsatellite polymorphisms in systemic lupus erythematosus: association with the anti-Sm immune response. Rheumatology 43: 1357-1363.

31. Sobkowiak A, Lianeri M, Wudarski M, Lacki JK, Jagodzinski PP (2009) Genetic variation in the interleukin-10 gene promoter in Polish patients with systemic lupus erythematosus. Rheumatol Int 29: 921-925.

32. Suarez A, Lopez P, Mozo L, Gutierrez C (2005) Differential effect of IL10 and TNF{alpha} genotypes on determining susceptibility to discoid and systemic lupus erythematosus. Ann Rheum Dis 64: 1605-1610.

33. Sung YK, Park BL, Shin HD, Kim LH, Kim SY, et al. (2006) Interleukin-10 gene polymorphisms are associated with the SLICC/ACR Damage Index in systemic lupus erythematosus. Rheumatology 45: 400-404.

34. Yu HH, Liu PH, Lin YC, Chen WJ, Lee JH, et al. (2010) Interleukin 4 and STAT6 gene polymorphisms are associated with systemic lupus erythematosus in Chinese patients. Lupus 19: 1219-1228.

35. Zhu LJ, Liu ZH, Zeng CH, Chen ZH, Yu C, et al. (2005) Association of interleukin-10 gene -592 A/C polymorphism with the clinical and pathological diversity of lupus nephritis. Clin Exp Rheumatol 23: 854-860.

36. Lan Y, Qin J, Wu J, Tang XS, Qin JM (2007) Association between the polymorphism in interleukin-10 gene promoter and systematic lupus erythematosus in a Chinese Zhuang population in Guangxi Zhuang Nationality Autonomous Region. Chinese Journal of Dermatology (in Chinese) 40(9) 555-557: 555-557.

37. Ren XY, Li Y, Ren LL, Wang L, Wu YK (2011) The research of genetic association of interleukin-10 polymorphisms with systemic lupus erythematosus. Guide of China Medicine (in Chinese) 9: 24-27.

38. Shen N (2003) Variations in the IL -10 promoter region confer susceptibility to systemic lupus erythematosus in Chinese population. Shanghai Yi Xue (in Chineses) 26: 452-459.

39. Wang FY, Li GC, Xie HF, Luo QZ, Zha GZ, et al. (2007) Polymorphism and SLE in Chinese Han population of Hunan province. Acta Laser Biology Sinica (in Chinese) 16: 344-348.

40. Xu AP, Lv J, Bai J, Liang YY, Lai DY, et al. (2007) Association of interleukin-10 gene polymorphism with systemic lupus erythematosus and lupus nephritis. Chinese Journal of Nephrology (in Chinese) 23: 429-433.

41. Zhou H, Qu R, Tang ZH, Liu C (2007) Investigation of the correlation between interleukin-10 promoter polymorphisms and systemic lupus erythematosus. Chinese Journal of Dermatology and Venereology (in Chineses) 21: 517-520.
